# Carbon Fiber-Reinforced Polyetheretherketone (PEEK) vs. Titanium Plates for Upper Limb Fractures: A Systematic Review

**DOI:** 10.7759/cureus.101094

**Published:** 2026-01-08

**Authors:** Ward Hamsho, Muhammad Y Raufi, Mohammad Alnajjar, Mahmoud Rhodes, Usman Hafeez, Rafya Aurangzeb

**Affiliations:** 1 Trauma and Orthopaedics, Leeds Teaching Hospitals NHS Trust, Leeds, GBR; 2 Medicine, LAK Locums, Leeds, GBR

**Keywords:** carbon-fiber–reinforced polyetheretherketone (cfr-peek), distal end radius fracture, proximal humeral fracture, titanium plates, upper limb fractures

## Abstract

Carbon fiber-reinforced polyetheretherketone (CFR-PEEK) plates have emerged as alternatives to titanium locking plates for fixation of upper-limb fractures. Advantages may include an elastic modulus closer to bone, fewer imaging artefacts, and reduced stress shielding. This systematic review synthesizes comparative clinical evidence. We analyzed data from five comparative studies (n = 210 patients; CFR-PEEK = 108, titanium = 102) provided in the dataset. Searches of PubMed and the Cochrane Library identified relevant trials. Data were extracted for demographics, interventions, functional outcomes, complications, and imaging findings, following PRISMA-style methodology. Five studies (three proximal humerus, two distal radius) were included. Overall complication rates were lower with CFR-PEEK compared to titanium. Functional outcomes were generally comparable, though one study showed improved Constant and OSS scores for CFR-PEEK. Imaging clarity was consistently superior with CFR-PEEK. CFR-PEEK plates demonstrate similar or superior clinical outcomes compared with titanium plates in upper limb fractures, with fewer complications and superior radiographic visibility. Evidence remains limited, requiring larger RCTs.

## Introduction and background

Upper limb fractures, particularly proximal humerus and distal radius fractures (DRFs), are common injuries that can lead to substantial functional impairment if not appropriately managed [1]. Stable fixation with plates remains the cornerstone of surgical treatment [2]. Although titanium locking plates are widely used and generally associated with satisfactory outcomes, their use is not without limitations. Reported complication rates for proximal humerus fractures (PHFs) treated with titanium plates can be as high as 30%, encompassing problems such as secondary screw perforation, loss of reduction, and implant-related irritation [3-6].

A key factor underlying these complications is the high rigidity of titanium implants, which may contribute to stress shielding and failure at the bone-screw interface, particularly in osteoporotic bone [7]. In addition, the metallic nature of titanium plates produces radiological artifacts, hindering the accurate assessment of fracture healing and complicating the early detection of postoperative issues [5].

Carbon fiber-reinforced polyetheretherketone (CFR-PEEK) plates have emerged as a potential alternative, offering several theoretical advantages. Their radiolucency allows for improved visualization of fracture alignment and healing on plain radiographs, while also minimizing artifact on MRI, thereby facilitating better assessment of surrounding soft tissues. Biomechanically, their bone-like elasticity reduces the risk of stress shielding and bone loss, and their material properties prevent cold welding between plates and screws, simplifying implant removal. Furthermore, CFR-PEEK is highly biocompatible, avoids metal hypersensitivity, and may even demonstrate osteoinductive potential [8,9].

Nevertheless, drawbacks remain. The higher cost and lack of intraoperative contouring currently limit the widespread adoption of CFR-PEEK plates in clinical practice [10]. The central question, therefore, is whether the theoretical and biomechanical advantages of CFR-PEEK implants translate into superior clinical outcomes compared with titanium plates.

Although early biomechanical and clinical studies have evaluated CFR-PEEK plates, a comprehensive synthesis of the available evidence is still lacking. To address this gap, the present systematic review aims to critically evaluate and compare the clinical efficacy, safety, and functional outcomes of CFR-PEEK vs. titanium plates in the surgical fixation of distal radius and PHFs. This review seeks to provide orthopedic surgeons with evidence-based insights to guide implant selection and optimize patient outcomes.

## Review

Review and methods

This systematic review was conducted in accordance with the Preferred Reporting Items for Systematic Reviews and Meta-Analyses (PRISMA) guidelines [11]. The review protocol was established as a priority to ensure methodological rigor and transparency.

Search Strategy

A comprehensive literature search was conducted on MEDLINE, Embase, CINAHL, PubMed, Google Scholar, and CENTRAL as of 14 September 2025. The search strategy included terms and keywords related to the fracture types, implant materials, and surgical interventions. The exact search query used was: ("proximal humerus" OR "distal radius") AND ("CFR-PEEK" OR "Carbon-Peek " OR "carbon fiber reinforced polyetheretherketone") AND ("titanium" OR "metal" OR "locking" OR "plate" OR "proximal humerus internal locking" OR "non-contact bridging") The search was not restricted by publication date or language.

Eligibility Criteria

Inclusion criteria: The inclusion criteria are the following: studies comparing CFR-PEEK plates with titanium plates for the fixation of distal radius or PHFs; studies reporting clinical outcomes (e.g., functional scores), radiographic outcomes (e.g., union rates, malunion), or complication rates (e.g., screw perforation, infection, hardware removal); and prospective or retrospective cohort studies, randomized controlled trials (RCTs), and comparative case series. Studies involving adult human participants.

Exclusion criteria: The exclusion criteria are the following: in vitro studies, biomechanical studies without clinical data, animal studies, cadaveric studies, and expert opinions; case reports, review articles, editorials, and conference abstracts without full-text availability; and studies not providing comparative data between CFR-PEEK and titanium plates.

Study Selection

Following the database searches, all identified records were imported. Duplicate records were removed. Two independent reviewers screened the titles and abstracts of the remaining records against the predefined eligibility criteria. Full-text articles of potentially relevant studies were then retrieved and independently assessed for eligibility. Any discrepancies during the screening and full-text review process were resolved through discussion.

**Figure 1:**
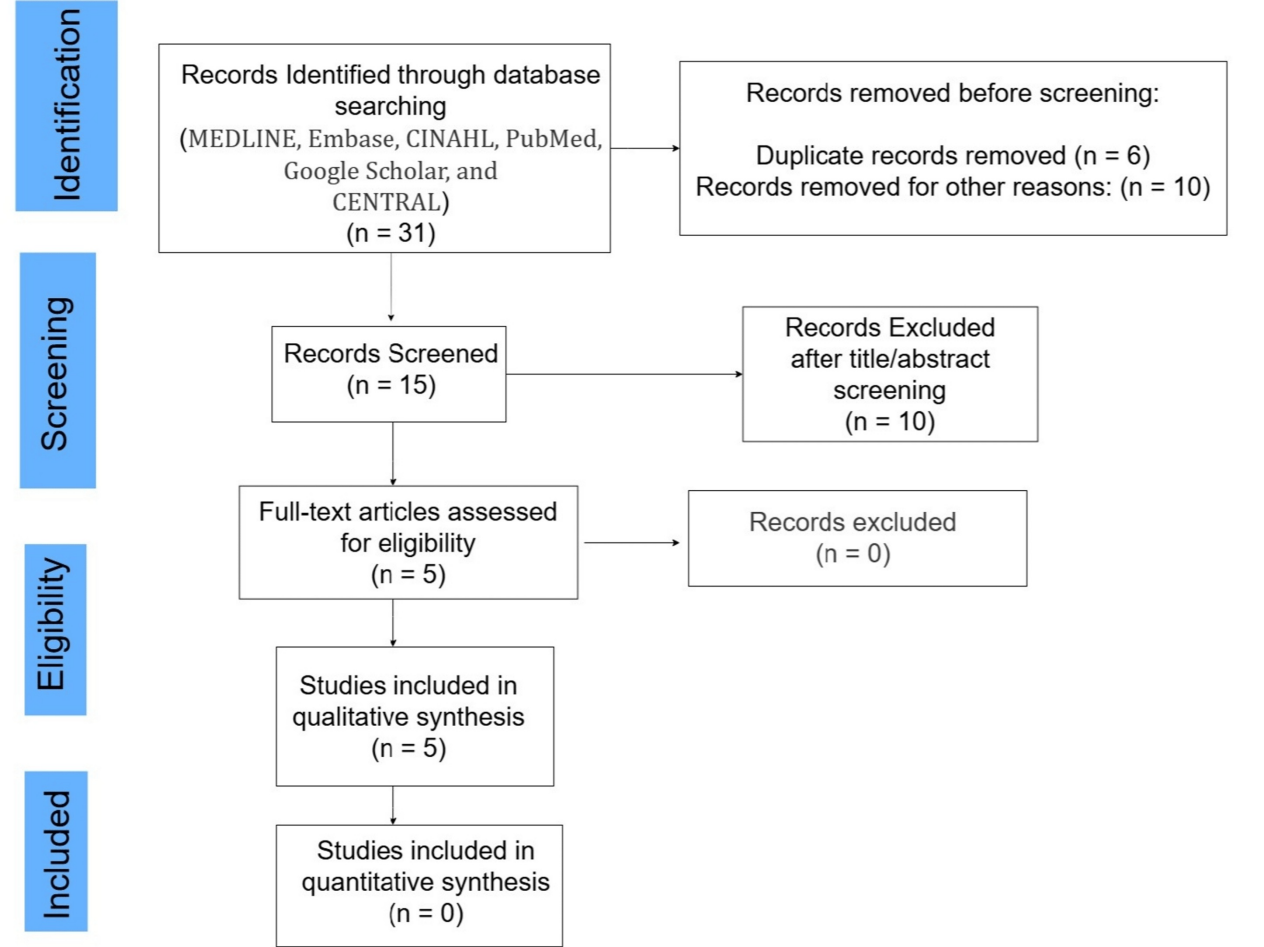
Preferred Reporting Items for Systematic Reviews and Meta-Analyses (PRISMA) flow chart

The PRISMA flow chart (Figure 1) illustrates the study selection process.

Data Extraction

Data from the included studies were extracted into a pre-designed Excel spreadsheet. The extracted data included the following: study characteristics - author, publication year, country, study design (e.g., RCT, cohort); patient demographics - number of patients in each group (CFR-PEEK and titanium), mean age, percentage of female participants; intervention details: fracture type (proximal humerus or distal radius), follow-up duration; outcomes - functional scores (e.g., Constant-Murley Score (CMS), Oxford Shoulder Score (OSS), Simple Shoulder Test (SST), Disability of Arm, Shoulder, and Hand (DASH), Mayo Wrist Score, Visual Analog Scale (VAS) for pain), union rates, non-union, malunion, infection rates, implant failure, hardware removal rates, and operative time; and complications: total complications, specific complications such as screw perforation, varus displacement, and avascular necrosis.

Risk of Bias Assessment

The methodological quality and risk of bias of the included studies were independently assessed by two reviewers using appropriate tools. For cohort studies, the Newcastle-Ottawa Scale (NOS) was utilized (Table 1), evaluating studies based on three broad perspectives: selection of the study groups, comparability of the groups, and ascertainment of either the exposure or outcome of interest [12]. For case series, the Joanna Briggs Institute (JBI) Critical Appraisal Checklist for Case Series was employed (Table 2) [13]. For RCTs, the Cochrane Collaboration's Tool for Assessing Risk of Bias in Randomized Trials was used (Table 3) [14]. Studies were categorized as having low, moderate, or high risk of bias based on the assessment. The risk of bias assessment results is summarized in the provided tables.

**Table 1:** Newcastle-Ottawa score for observational studies

Study	Selection (max 4)	Comparability (max 2)	Outcome (max 3)	Total (max 9)	Risk of bias
Schliemann et al. 2015 - proximal humerus [15]	4	1	3	8/9	Low-moderate
Katthagen et al. 2017 - proximal humerus [16]	4	1	3	8/9	Low-moderate
Behrendt et al. 2017 - distal radius [17]	4	1	2	7/9	Moderate

**Table 2:** Joanna Briggs Institute (JBI) critical appraisal checklist for case series [16]

Domain	Assessment	Notes
Inclusion criteria	Low risk	Clear eligibility (displaced unilateral PHF, ≥18 years, consent).
Case identification	Low risk	Surgical records, prospective enrolment.
Consecutive inclusion	Low risk	All eligible between April 2013 and February 2014 included (except two exclusions).
Demographics and clinical info	Low risk	Age, sex, fracture classification, and operative details reported.
Outcome measurement	Low risk	Functional scores (constant, SST, SSV, and VAS), radiology, complications.
Follow-up completeness	Low risk	95% follow-up at 12 months.
Statistical analysis	Low risk	Appropriate non-parametric tests for small cohort.
Overall	Low risk of bias	Well-reported, but small single-center cohort and short (12-month) follow-up limit external validity.

**Table 3:** Risk of bias assessment for randomized controlled trials utilizing the Cochrane Collaboration's tool

First author	Bias	Authors judgement	Support for judgment
Ziegler et al. 2023 [18]	Random sequence generation (selection bias); allocation concealment	Low risk	A randomization list was generated (Excel random numbers) and results were put into consecutively numbered envelopes opened by the operating surgeon immediately before surgery.
	Selective reporting (reporting bias)	Low risk	Trial was registered, outcomes described in the paper match the reported endpoints and sensitivity analyses are reported.
	Other bias; other sources of bias		No other bias detected.
	Blinding of participants and personnel (performance bias)	Unclear	Blinding is not clearly stated.
	Blinding of outcome assessment (detection bias)	High risk	Primary outcomes are patient-reported functional scores. Assessors/patients were not blinded, increasing risk that subjective measures could be influenced by knowledge of implant type.
	Incomplete outcome data (attrition bias)	High risk	Substantial attrition by 12 months: of 76 randomized, 54 had one-year data (follow-up rate ~71%); 22 lost/excluded
Perugia et al. 2017 [19]	Random sequence generation (selection bias); allocation concealment	Unclear	Sequence generation was computer-generated. Allocation concealment details are not reported, so we cannot be confident concealment was secure.
	Selective reporting (reporting bias)	Low risk	Prespecified outcomes are reported.
	Other bias; other sources of bias		No other bias detected.
	Blinding of participants and personnel (performance bias)	Unclear	Blinding is not clearly stated.
	Blinding of outcome assessment (detection bias)	Unclear	Blinding is not clearly stated.
	Incomplete outcome data (attrition bias)	Low risk	The paper reports no losses to follow-up at the reported timepoints.

Data Synthesis

Due to high heterogeneity in study designs, outcome measures, and reporting formats, a meta-analysis was not performed. Instead, a narrative synthesis of the findings was conducted. Data were summarized descriptively, highlighting key characteristics, outcomes, and complication rates for both CFR-PEEK and titanium plate groups. Comparisons were made qualitatively, focusing on trends and significant differences reported by individual studies.

Results

Included Studies

The systematic search and screening process identified five studies that met the inclusion criteria: Schliemann et al. [15], Katthagen et al. [16], Behrendt et al. [17], Ziegler et al. [18], and Perugia et al. [19].

Patient Demographics and Study Characteristics

Three studies (Schliemann et al. [15]; Katthagen et al. [16]; Ziegler et al. [18]) evaluated PHFs, while two (Behrendt et al. [17]; Perugia et al. [19]) investigated DRFs. Across all studies, 108 patients were treated with CFR-PEEK plates and 102 with titanium plates. Mean ages ranged from 56.8 to 66.8 years in the CFR-PEEK groups and 52.6 to 67.4 years in the titanium groups, representing a predominantly older population. Follow-up periods ranged from 12 to 24 months (Table 4).

**Table 4:** Demographics and study characteristics CFR-PEEK, carbon-fiber-reinforced polyetheretherketone; DR, distal radius; PH, proximal humerus; RCT, randomized controlled trial; Ti, titanium

Serial Number	Author	Year	Country	Design	Fracture	Patients CFR-PEEK (n)	Patients Ti (n)	Age CFR-PEEK	Age Ti	% Female CFR-PEEK	% Female Ti	Follow-up in months
1	Schliemann et al. [15]	2015	Germany	Prospective cohort	PH	29	29	66.4	66.4	NR	NR	24
2	Katthagen et al. [16]	2017	Germany	Case series	PH	21	21	66.8	67.4	66.7	66.7	12
3	Behrendt et al. [17]	2017	Germany	Prospective cohort	DR	14	12	57.2	61.7	80	79	6-12
4	Ziegler et al. [18]	2023	Germany	RCT	PH	29	25	62.5	62.8	82.8	84.0	12
5	Perugia et al. [19]	2017	Italy	RCT	DR	15	15	56.8	52.6	66.7	73.3	16.1 average

Functional Outcomes

Proximal humerus fractures: Schliemann et al. [15] conducted a prospective matched cohort study comparing 29 CFR-PEEK and 29 titanium fixations, demonstrating significantly superior outcomes for CFR-PEEK at 24 months. The CMS was higher in the CFR-PEEK group (71.3 vs. 59.2; p = 0.038), as were the OSS (27.4 vs. 21.6; p = 0.029) and Simple Shoulder Test (59% vs. 48%) [15]. Katthagen et al. [16], in a prospective case-control study (21 CFR-PEEK vs. 21 titanium), found no significant differences between groups at 12 months. Mean Constant Score was 73.8 ± 15 (CFR-PEEK) vs. 69.4 ± 18.5 (titanium), SST was 9.3 ± 2.6 vs. 8.7 ± 2.9, and Simple Shoulder Value (SSV) was 74% vs. 72% [16]. Similarly, Ziegler et al. reported no significant differences in functional outcomes at 12 months in their prospective RCT (29 CFR-PEEK vs. 25 titanium; p ≈ 0.57). DASH scores were 18.6 ± 14.7 (CFR-PEEK) vs. 23.9 ± 22.0 (titanium), with comparable OSS and SST results [18].

Distal radius fractures: Behrendt et al. (2017) conducted a prospective clinical pilot study (14 CFR-PEEK vs. 12 titanium) that demonstrated comparable clinical and radiographic outcomes between groups. At 12 months, follow-up data were available for five CFR-PEEK patients, showing a mean DASH score of 5.3 ± 9.5 and a Mayo Wrist Score of 83.6 ± 9.5 [17]. The intergroup comparison at six weeks, presented graphically, indicated a mean DASH score of approximately 25 in the CFR-PEEK group vs. 32-33 in the titanium group, suggesting slightly better early functional outcomes with CFR-PEEK fixation, though not statistically significant (p > 0.05). Overall, while long-term titanium data were not reported, early results suggested a trend toward improved range of motion and patient satisfaction in the CFR-PEEK group [17]. Similarly, Perugia et al. [19], in an RCT (15 CFR-PEEK vs. 15 titanium; mean follow-up 16.1 months), observed no significant intergroup differences. DASH scores were 15.3 (CFR-PEEK) vs. 13.2 (titanium; p > 0.05), with comparable radiographic and VAS outcomes [19].

Complications

Proximal humerus fractures: Schliemann et al. reported a lower overall complication rate in the CFR-PEEK group (21%) compared to titanium (38%). Varus malunion occurred in 14% (CFR-PEEK) vs. 24% (titanium), while avascular necrosis was observed in 3% vs. 10%, respectively [15]. Katthagen et al. [18] found significant differences in implant-related complications, with none occurring in the CFR-PEEK group, whereas five cases of screw perforation requiring revision were noted in the titanium group (p = 0.048). Four non-implant-related revisions (19%) occurred in the CFR-PEEK group [16]. Ziegler et al. (2023) did not explicitly report complication data [18].

Distal radius fractures: Behrendt et al. [17] observed no implant-related complications or failures in the CFR-PEEK group. In contrast, two intraoperative screw head damages occurred in the titanium group, requiring replacement but not classified as implant failures. No non-union, malunion, or infection cases were reported in either group [17]. Perugia et al. also reported no implant-related complications in either the CFR-PEEK or titanium cohorts (see Table 5 for detailed outcomes and complications) [19].

**Table 5:** Outcomes and complications CFR-PEEK, carbon-fiber-reinforced polyetheretherketone; CMS, Constant-Murley Score; DASH, Disabilities of the Arm, Shoulder and Hand; NR, not reported; SST, Simple Shoulder Test; Ti, titanium; VAS, visual analog score

Study (author, year)	Functional Score (CFR-PEEK vs. Ti)	Complications CFR-PEEK	Complications Ti	Non-union CFR-PEEK	Non-union Ti	Malunion CFR-PEEK (n)	Malunion Ti (n)
Schliemann et al., 2015 [15]	CMS 71.3 (44-97) vs. 59.2 (28-86); p = 0.038	6/29 (21%)	11/29 (38%)	0	0	4 (14%)	7 (24%)
Katthagen et al., 2017 [16]	CMS 73.8 ± 15 vs. 69.4 ± 18.5; p = 0.43	0/21	5/21	0	0	0	3/5
Behrendt et al., 2017 [17]	DASH at 6 weeks ~25 vs. ~32-33 (p > 0.05); DASH at 12 months 5.3 ± 9.5 vs. no data at 12 months for Ti	0	2	0	0	0	0
Ziegler et al., 2023 [18]	DASH 18.6 ± 14.7 vs. 23.9 ± 22.0; p ≈ 0.57	NR	NR	NR	NR	NR	NR
Perugia et al., 2017 [19]	DASH 15.3 (2.5-58.9) vs. 13.2 (10.6-54.8); p > 0.05	0	0	0	0	0	0

Other Outcomes

Operative time: Behrendt et al. [17] reported comparable operative durations between groups, with mean times of 101 ± 18.6 minutes for CFR-PEEK and 109.3 ± 10.5 minutes for titanium, showing no statistically significant difference. Operative times were not reported in the other included studies.

Hardware removal and radiolucency: Schliemann et al. [15] documented hardware removal in 24% (7/29) of CFR-PEEK patients and 28% (8/29) of titanium patients. Katthagen et al. [16] noted four hardware removals in the CFR-PEEK group, all unrelated to implant failure, and five removals in the titanium group, each associated with revision surgery for screw perforation. All included studies highlighted the clinical advantage of CFR-PEEK plates’ radiolucency, which facilitated improved postoperative imaging and assessment of fracture healing while eliminating the radiographic and MRI artifacts typically associated with metallic implants.

Discussion

This systematic review sought to compare the clinical outcomes, complication rates, and functional results of CFR-PEEK plates vs. conventional titanium plates in the fixation of distal radius and PHFs. Across five comparative studies, our findings indicate that CFR-PEEK plates provide outcomes that are at least comparable to titanium plates, with potential advantages in reducing implant-related complications and improving postoperative radiographic assessment.

One of the most notable findings was the lower incidence of implant-related complications, particularly screw perforations, in the CFR-PEEK groups for PHFs. Katthagen et al. [16] demonstrated a significantly higher rate of screw perforations requiring revision in the titanium group compared to the CFR-PEEK group. This aligns with the theoretical biomechanical advantages of CFR-PEEK, whose elastic modulus more closely approximates that of cortical bone. By enabling more physiological load transfer at the bone-implant interface, CFR-PEEK is thought to reduce stress shielding and the risk of screw penetration, particularly in osteoporotic bone, which is common in these patient populations [8,9]. In contrast, the rigidity of titanium plates, while advantageous for achieving stable fixation, may concentrate stress at the screw tips, predisposing to perforations and other implant-related complications.

Functional outcomes were largely equivalent between CFR-PEEK and titanium plates. Schliemann et al. reported significantly higher Constant-Murley and OSS at 24 months in the CFR-PEEK group, suggesting potential long-term benefits [15]. However, Katthagen et al. and Ziegler et al. observed no significant differences at 12 months, indicating comparable efficacy in the shorter term [16,18]. For DRFs, Behrendt et al. and Perugia et al. both reported similar clinical and radiological outcomes, with early trends suggesting improved motion and satisfaction with CFR-PEEK [17,19]. Collectively, these findings suggest that CFR-PEEK is non-inferior to titanium in restoring function and may offer selective benefits in specific contexts or over longer follow-up periods.

The radiolucency of CFR-PEEK implants emerged as a consistent advantage across all studies [15-17]. This feature facilitates superior postoperative imaging, enabling clearer visualization of fracture healing and implant positioning, without the artifacts that limit titanium plates on radiographs or MRI. Improved imaging can support earlier detection of complications and enhance clinical decision-making during follow-up. Operative times were reported as similar between groups [17], suggesting no added technical burden when using CFR-PEEK. Hardware removal rates were also comparable, though it is noteworthy that removals in the titanium groups were often necessitated by implant-related issues such as screw perforation, whereas removals in the CFR-PEEK groups were generally unrelated to implant failure [16].

Comparison With Existing Literature

These findings are consistent with the growing body of evidence supporting CFR-PEEK technology in orthopaedic applications. Biomechanical studies have previously highlighted the favourable mechanical properties of CFR-PEEK, particularly its bone-like elasticity, which mitigates stress shielding and reduces implant-bone interface complications [8]. 

Implications for Clinical Practice

The results of this review suggest that CFR-PEEK plates represent a viable, and in some respects advantageous, alternative to titanium plates for fixation of proximal humerus and DRFs. Surgeons may particularly consider CFR-PEEK in patients at high risk of implant-related complications, such as screw perforation, or in situations where high-quality postoperative imaging is essential. Implant choice should, however, remain individualized, accounting for fracture pattern, bone quality, and implant design features, including the availability of polyaxial screw placement in certain systems.

Limitations

This review has several limitations. The total number of available comparative studies remains small, and all were conducted in European populations, which may limit generalizability. Although two RCTs were included [18,19], the remaining studies were observational, and even the RCTs had methodological limitations, including small sample sizes, potential attrition bias, and relatively short follow-up. Heterogeneity in fracture types (proximal humerus vs. distal radius) and outcome measures precluded a formal meta-analysis. Moreover, the maximum follow-up duration across studies was 24 months, which may not fully capture long-term complications, such as late implant failures or degenerative changes. Despite these limitations, this review synthesizes the best available comparative evidence and identifies clear trends favoring CFR-PEEK in certain outcome domains.

## Conclusions

In summary, CFR-PEEK plates appear to be a safe and effective alternative to titanium plates for the fixation of proximal humerus and DRFs. While functional outcomes are generally comparable, CFR-PEEK demonstrates a clear advantage in reducing implant-related complications, particularly screw perforations, and provides superior postoperative radiographic visualization. These findings have practical implications for implant selection, especially in patients with osteoporotic bone or where radiological clarity is critical for follow-up.

Nonetheless, the evidence base remains limited, and larger, multicenter RCTs with extended follow-up are required to validate these findings, assess cost-effectiveness, and define the specific patient subgroups most likely to benefit. Future research should also explore long-term functional outcomes and implant survivorship, thereby informing evidence-based guidelines for implant choice in upper-limb fracture fixation.
